# Heterogeneity in global vegetation and terrestrial climate change during the late Eocene to early Oligocene transition

**DOI:** 10.1038/srep43386

**Published:** 2017-02-24

**Authors:** Matthew J. Pound, Ulrich Salzmann

**Affiliations:** 1 Department of Geography, Northumbria University, Newcastle upon Tyne, NE1 8ST, UK

## Abstract

Rapid global cooling at the Eocene – Oligocene Transition (EOT), ~33.9–33.5 Ma, is widely considered to mark the onset of the modern icehouse world. A large and rapid drop in atmospheric pCO_2_ has been proposed as the driving force behind extinctions in the marine realm and glaciation on Antarctica. However, the global terrestrial response to this cooling is uncertain. Here we present the first global vegetation and terrestrial temperature reconstructions for the EOT. Using an extensive palynological dataset, that has been statistically grouped into palaeo-biomes, we show a more transitional nature of terrestrial climate change by indicating a spatial and temporal heterogeneity of vegetation change at the EOT in both hemispheres. The reconstructed terrestrial temperatures show for many regions a cooling that started well before the EOT and continued into the Early Oligocene. We conclude that the heterogeneous pattern of global vegetation change has been controlled by a combination of multiple forcings, such as tectonics, sea-level fall and long-term decline in greenhouse gas concentrations during the late Eocene to early Oligocene, and does not represent a single response to a rapid decline in atmospheric pCO_2_ at the EOT.

The Eocene – Oligocene (EOT) transition (ca. 33.9–33.5 Ma) is widely considered to be the biggest step in Cenozoic climate evolution associated with a global cooling, disruption of marine ecosystems and the onset of Antarctic glaciation^1^,^2^,^3^,^4^,^5^,^6^. A tectonic opening of the Southern Ocean gateways, as well as a decline in global atmospheric CO_2_ concentrations, have been discussed as the potential main drivers for this greenhouse to icehouse transition^1^,^2^,^3^,^4^,^5^,^6^. The tectonic opening of the ocean gateways in the Tasmanian Sea and Drake Passage establishing the Antarctic Circumpolar Current (ACC) and thermal isolation of Antarctica have long been proposed as the major driver for global climate change at the EOT^1^,^7^. However, geological evidence from marine proxies and climate model studies have challenged this hypothesis of a southern hemisphere driven, tectonically controlled global climate change on the grounds of a potential time lag between the onset of the ACC and palaeogeographic changes^8^,^9^. Climate modelling studies and boron isotope studies identified falling atmospheric pCO_2_ levels at the EOT as potentially being the primary driver for ice sheet growth and cooling, suggesting the ACC may have been of secondary importance^10^,^11^. Estimates of atmospheric pCO_2_ concentrations using boron isotopes from a geological section in Tanzania identified a drop of 600 ppmv (1100 to 500 ppmv) over a period of 0.4 Ma at the EOT^4^ (Fig. 1). Using alkenone proxies in combination with climate modelling, Pagani *et al*.^5^ estimated that atmospheric pCO_2_ fell at the EOT by 40% over three million years, whereas reconstructions from the stomata of leaves preserved in sediments of central Germany indicate continuously decreasing atmospheric CO_2_ concentration during the Late Eocene that reached a stable low value before the EOT^12^ (Fig. 1).

The EOT is represented in the marine realm by a two stepped shift in the bottom water oxygen isotopes associated with glacial advance on Antarctica^2^,^13^, extinctions in the marine realm^1^, a deepening of the calcium compensation depth^14^ and a cooling of global climate^2^,^6^. The persistence of this global cooling is uncertain, with some records suggesting a return to near-Eocene climate shortly after the EOT^3^,^15^,^16^, whilst others show that the EOT was a permanent cooling step^2^,^6^. The argument of climatic rebound versus climate step are further compounded by a growing body of recent work that suggests the benthic oxygen isotope signal can be influenced by oceanic circulation changes without atmospheric cooling^7^ and that the palaeotopography of Antarctica could have held a larger ice sheet, thus requiring less of the oxygen isotope signal to be from temperature^17^.

The discussion about forcing and timing of climate change at the EOT is primarily based on evidence from model simulations and geochemical proxies derived from marine sediment cores. Some terrestrial oxygen isotope records appear to support the marine evidence by showing up to 8 °C lower mean annual temperatures (MAT) in the mid-latitudes of the northern hemisphere after the EOT^18^,^19^. Here we use a global synthesis of pollen and spore assemblages from 216 Priabonian (38–33.9 Ma) to Rupelian (33.9–28.1 Ma) geological sites (Fig. 2), to test whether the distinct marine cooling signal at the EOT coincides with a major global reorganisation of vegetation zones on land. Previous palaeobotanical studies have shown that global vegetation throughout the Cenozoic responded in a highly sensitive manner to fluctuations in climate and atmospheric CO_2_ concentrations with major shifts of vegetation zones and changes in species composition^20^,^21^. We hypothesise that a global drop in atmospheric pCO_2_ at the EOT in the order of 400–600 ppmv^4^,^5^ must have resulted in a strong global terrestrial cooling, and subsequent simultaneous shift of latitudinal vegetation zones in both hemispheres. Our study did not find such a strong equatorward shift in palaeo-biomes or terrestrial cooling and therefore challenges previous reconstructions of a large and rapid pCO_2_ decline at the EOT.

## Results

### The terrestrial record

For the terrestrial realm, the lack of long and well dated sediment records puts considerable constraints on a detailed spatial and temporal reconstruction of environmental change at the EOT. The terrestrial proxy dataset crossing the EOT is very sparse and evidence seems to be less clear than in the marine records with no consistent pattern of change^1^. The palaeoecological evidence can be divided into three major groups (Fig. 2) which show either **(a)** no change across the EOT^22^,^23^, (**b)** a pronounced cooling at the EOT^17^,^24^,^25^,^26^, often associated with an increase in seasonality and lower winter temperatures^27^,^28^, or (**c)** a gradual change that had already started before the EOT during the middle to late Eocene and continued into the Oligocene^12^,^23^,^29^,^30^. Terrestrial oxygen isotopes have shown no change in South America^31^, but a strong cooling in North America and northwest Europe^18^,^19^. Mammal faunal turnovers at the EOT are well documented on Eurasia^24^, but do not seem to have occurred in North America, where tooth mesowear from mammalian herbivores shows long-term change from the Middle Eocene rather than short-term change at the EOT^32^.

### Pre and post EOT: comparison of Priabonian and Rupelian biome distribution

From 216 localities with palynological data (59 localities with samples for both the Priabonian and Rupelian), 154 Priabonian and 106 Rupelian palaeo-biomes have been reconstructed. The statistically defined palaeo-biomes show a latitudinal distribution that is representative of a world much warmer than today with warm-temperate forests reaching the Arctic Circle. A comparison between the Rupelian and Priabonian biome distribution reveals a mixed picture of regional change and no change (Fig. 3). Following guidance developed by the Intergovernmental Panel on

Climate Change, we qualitatively assessed the level of confidence of biome and temperature reconstruction for each site^21^,^33^. No changes in palaeo-biome distribution between the Priabonian and Rupelian can be detected in South America, Africa or Australia. In South America the tropical evergreen forest biomes have strongly varying confidence levels, which are related to the dating accuracy of these records. Whilst the cool-temperate forest and cool-temperate shrublands of southern South America and the Antarctic Peninsula have low to medium-confidence due to limited dating controls and/or purely biostratigraphical focus of the respective study (Supplementary Table S2). During the Rupelian there is evidence for cool-temperate mixed forest around the Weddell Sea and the first evidence for tundra (high to very high-confidence) in east Antarctica at 75°S. Palynological data from the Priabonian are restricted to 60–68°S and show the presence of cool-temperate mixed forest and cool-temperate shrubland in Prydz Bay and around the Weddell Sea (Fig. 3). In North America, the geographical distribution of palaeo-biomes only changes in the high-latitudes, where in the Rupelian a southward shift of the northernmost limit of warm-temperate mixed forest of approximately 5 degrees is recorded from a single low-confidence site (Fig. 3). A similar response, indicating a potential cooling at the EOT, can be reconstructed for the North Atlantic region where a low-confidence record of paratropical/subtropical mixed forest extended north to 65.8°N during the Priabonian, but only to 56.7°N in the Rupelian (Fig. 3). In Europe, palaeo-biomes representative of seasonal climates are restricted to the Iberian Peninsula and western France during the Priabonian (Fig. 3); by the Rupelian these had expanded eastwards along the Mediterranean coast of France and there was the first occurrence of xerophytic shrubland on the coast of the central Mediterranean (high-confidence locality). In central Asia vegetation changed from Priabonian warm-temperate mixed forests to paratropical/subtropical mixed forests in the Rupelian at 61–65°N; this is the only locality of the 59 with both a Priabonian and Rupelian pollen assemblage to show a change in palaeo-biome (Supplementary Table S1). Whereas the opposite change was recorded in East Asia at 49°N (medium-confidence localities due to low dating resolution).

### Changes in pollen assemblages across the EOT

The new statistically defined palaeo-biomes (Fig. 3) represent “snapshots” of land surface cover at a particular time during the Eocene and Oligocene. It is therefore possible that our Rupelian and Priabonian biome reconstructions could have missed climate change at the EOT, if it was not a major one-step change but rather a short-term cooling event that was followed by a return to warmer pre-EOT levels^16^. In order to capture such a transient short term event, we focussed our analyses on selected samples with a sufficient time-resolution across the EOT and applied the Jaccard Similarity coefficient to compare the similarity between each sample^34^ (Fig. 4). If the EOT caused a short term latitudinal shift in vegetation this will be reflected in the pollen assemblage at each of these sites through a decrease in the Jaccard Similarity coefficient. However, the Jaccard Similarity coefficient of pollen assemblages remains unchanged or increases, indicating equal or increased similarity of vegetation before, at and after the EOT in six out of eleven localities: southern Australia (N11), tropical South America (N9), the North Atlantic realm (N2, N3 in Fig. 4), southern North America (N7) and western Kamchatka (N4). In southern North America (N7) and North Atlantic (ODP 913, N3), the Jaccard Similarity of pollen assemblages decreases during the Late Eocene well before the EOT (Fig. 4). Decreasing Jaccard Similarity of pollen assemblages at ca 33.5 Ma are recorded in the Paris Basin (N8), central Asia (N6), and in the Labrador Sea (N1, N5), suggesting a change in vegetation at the EOT (Fig. 4). In the Paris Basin this change in similarity (Fig. 4) also coincides with decreasing pollen preservation due to taphonomic factors related to sea-level changes^35^.

### Quantitative estimates of climate change at the EOT

Our terrestrial climate reconstructions are based upon 16 regional groupings of 186 individual pollen assemblages to produce temporal temperature plots (Fig. 5; Supplementary Figures S2 and S3). Our regional terrestrial climate reconstructions are characterised by a complex pattern of pre-EOT cooling (four regions), cooling across the EOT (two regions and another three possible regions) and regions with no reconstructed change (five regions) (Fig. 5). Regions where Mean Annual Temperature Ranges (MATR) remain largely unchanged or do not show a clear cooling trend over the EOT include eastern North America (N2 in Fig. 5), North Atlantic region (N3), South America (N11; N13), Africa (N14), southeast Australia (N15) and tropical Asia and India (N16). In north eastern North America (N2), pre-EOT MATR was 17.0–20.8 °C, following the EOT MATR remained 17.0–20.8 °C with one record reconstructing 13.3–14.0 °C (Fig. 5). The North Atlantic region (N3) was 14.0–16.5 °C until 34.7 Ma when the MATR cooled to 11.6–14.0 °C (Fig. 5). After 34.7 Ma, MATR remained constant across the EOT until 32–31 Ma when a warming to 14–16.5 °C is reconstructed (Fig. 5). In tropical South America (N11; N13), tropical Africa (N14) and tropical Asia and India (N16) reconstructed MATR throughout the studied time interval was 18.0–27.5 °C (Fig. 5). However, our method is based on Nearest Living Relatives (NLR) and therefore not able to reconstruct tropical temperatures above modern values. In southeast Australia (N15) Priabonian MATR was 13.4–20.6 °C until ~36 Ma, after which the reconstructed MATR cooled to 11.3–15.5 °C (Fig. 5). This MATR remained relatively constant until 32–31 Ma when MATR increases to 12.2–20.6 °C (Fig. 5).

A climate cooling at the EOT can be reconstructed from Central Asia (N8) and Gulf Coast North America (N9). Pre-EOT reconstructed MATR in Central Asia (N8) was 17.0–22.2 °C, by 34 Ma this reconstructed MATR narrows to 19.7–21.9 °C, giving increased confidence to the climate reconstruction immediately before the EOT (Fig. 5). At the EOT the reconstructed MATR for Central Asia was 17.0–18.4 °C demonstrating a difference in MATR between the latest Priabonian and the EOT of −1.3 to −4.9 °C (Fig. 5). Reconstructed MATR for the Rupelian of Central Asia returns to a wider reconstructed range of 16.5–21.7 °C (Fig. 5). The Gulf Coast North America region (N9) has pre-EOT MATR reconstructions of 17.4–23.9 °C and MATR reconstructions at the EOT of 17.0–19.4 °C and 17.0–20.8 °C (Fig. 5) suggesting a cooling in the region of 0.4–4.5 °C. Reconstructed MATRs for the Rupelian are 18.4–19.7 °C, with only one low-confidence record of 13.3–23.9 °C possibly suggesting a return to pre-EOT MATRs (Fig. 5). Other regions which might show cooling across the EOT, but lack sufficient dating control, include northeast Asia (N6), western North America (N7), East Asia (N10) and southern West Atlantic (N12). In northeast Asia (N6) MATR appears to cool during the Priabonian from 18.3–19.7 °C to 13.8–14.0 °C, with temperatures at the EOT remaining at 13.8–14.0 °C (Fig. 5). However, all of these sites have low-confidence dating and as such it is difficult to know when the coldest MATR was reached (Fig. 5). The northeast Asia region also shows a post-EOT warming with reconstructed MATR reaching 16.1–17.0 °C (Fig. 5). Unfortunately, these sites continue to be low-confidence and as such it is unknown when exactly this warming occurred. Localities from western North America (N7) have a mixture of high to low-confidence dating, which makes identifying an interval of cooling complex (Fig. 5). Based on pollen assemblages with high-confidence dating it is possible to state that early Priabonian MATR was 16.4–20.8 °C, whilst MATR after 30 Ma is reconstructed as 11.6–18.4 °C (Fig. 5). In East Asia (N10) reconstructed MATRs for the Priabonian were 18.4–26.9 °C, whilst those for the Rupelian were 18.3–23.6 °C (Fig. 5). Whilst there is considerable overlap of Priabonian and Rupelian MATRs from western North America and East Asia, both regions show a reduction in both the minimum and maximum value of the reconstructed CA range. This would suggest a climatic cooling, but uncertainty in dating prevents us from attributing it to the EOT. In southern West Atlantic (N12) MATR before 35 Ma is reconstructed as 15.5–18.4 °C; after 35 Ma, and for the remainder of the Priabonian, MATR was between 13.4–15.5 °C (Fig. 5). After the EOT, MATR reconstructions for southern West Atlantic (N12) are 11.7–13.7 °C and 15.5–23.4 °C demonstrating a degree of uncertainty in Rupelian climate in this region (Fig. 5). Regions with a strong overlap of pre- and post EOT temperature ranges which do not allow identification of clear climate trends are northwest North America (N1), northwest Europe (N4) and the circum-Mediterranean (N5) (Fig. 5).

To identify potential seasonality changes across the EOT, we also reconstructed Cold Month Mean Temperature Ranges (CMMTR) and Warm Month Mean Temperature Ranges (WMMTR) for all regions (Supplementary Figures S2 and S3). The CMMTR and WMMTR show a similar heterogeneous pattern to the MATRs. The CMMT declines at the EOT in Central Asia (N8) and Gulf Coast North America (N9), with possible EOT cooling (high uncertainty due to overlapping temporal ranges) in western North America (N7), East Asia (N10) and western South Atlantic (N13). Pre-EOT cooling of CMMTR are reconstructed for three regions: North Atlantic (N3), northeast Asia (N6) and southeast Australia (N15). No change in CMMTR is reconstructed for northeast North America (N2), the Mediterranean (N5), South America (N11, N13), Africa (N14), tropical Asia and India (N16). A post-EOT cooling of CMMTR occurred in the Mediterranean (N5) at around 31 Ma, which returned to preceding CMMTR levels by 29 Ma (Supplementary Figure S2). Only two regions have changes in CMMTR at the EOT and three regions have low-confidence changes in CMMTR at the EOT, our results contrast with previous studies suggesting a global-scale cooling of winter temperatures^28^,^29^. A decline of WMMTR at the EOT occurred in Central Asia (N8) and the Gulf Coast of North America (N9), whilst low-confidence EOT WMMTR cooling may have happened in western North America (N7) and western South Atlantic (N12) (Supplementary Figure S3). Pre-EOT decline in WMMTR are reconstructed for four regions: North Atlantic (N3), Mediterranean (N5), northeast Asia (N6) and southeast Australia (N15). Unchanging WMMTR is reconstructed for northeast North America (N2), east Asia (N10), South America (N11, N13), Africa (N14) and tropical Asia and India (N16) (Supplementary Figure S3).

## Discussion

Climate change during the EOT has been linked to a decline in atmospheric CO_**2**_ of 400–600 ppmv over a 0.5 Ma period^4^,^5^, which should have forced a latitudinal reorganisation of biome distributions^36^. Global reconstructions of Neogene vegetation and terrestrial temperatures using similar methods to this study demonstrated the high-sensitivity of latitudinal biome distribution to past changes in global climate and CO_2_^20^,^21^. For example, a decline in atmospheric CO_2_ of ca. 150–250 ppmv from the end of the Mid-Miocene Climate Optimum to the Messinian resulted in a uniform equatorward shift of vegetation zones of more than 10 degrees latitude^20^. Climate sensitivity (the response of global temperature to a doubling of pCO_2_) might have been much lower during the Paleogene than during the Neogene^9^,^37^. However, as the temperature response to changes in pCO_2_ is strongly amplified at higher latitudes, an abrupt change of global temperature by even a few degrees should trigger a uniform shift of high latitude vegetation zones^20^,^21^,^36^.

**Figure 1:**
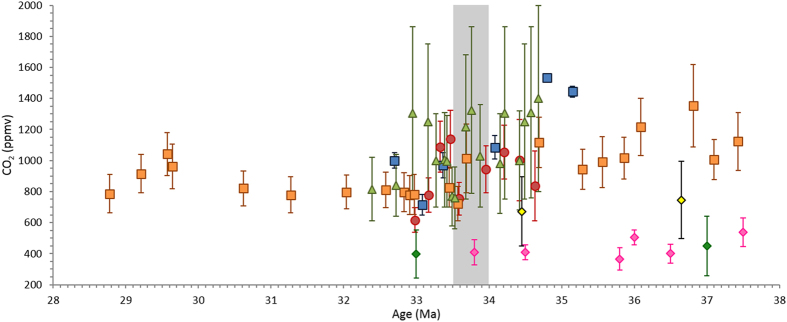
Compilation of published atmospheric pCO_2_ reconstructions crossing the EOT. Symbols denote different proxies: circles = boron isotopes (red^4^), diamonds = stomatal indices (yellow^99^; dark green^100^,^101^; pink^12^), squares = alkenones (blue^5^; orange^102^) and triangles = diatom carbon isotopes (green). The CO_2_ drawdown and minima associated with the EOT is located at 33.7–33.5 Ma in the Pearson *et al*.^4^, Zhang *et al*.^102^ and Heureux and Rickaby^103^ records.

**Figure 2:**
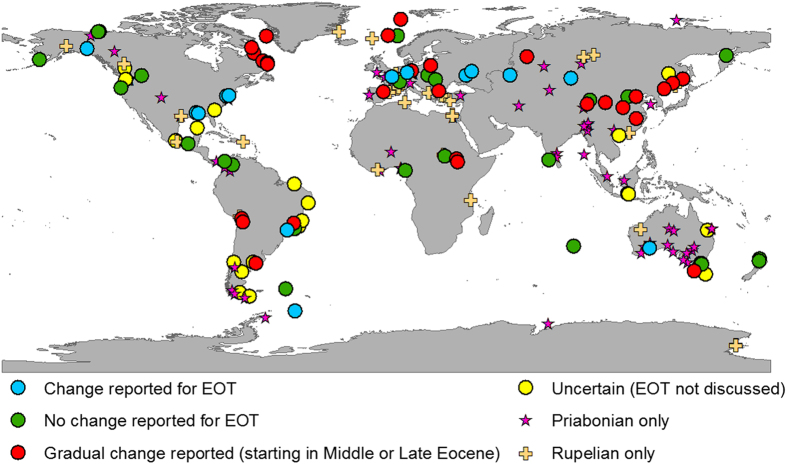
Selected 216 palynological sites covering the EOT. Blue circles show sites where the original author’s interpretation state either a change in flora or climate having occurred at the EOT (N = 14). Green circles denote palynological sites where no change in either flora and/or climate occurred at the EOT (N = 27). Red circles show the location of sites with a gradual change (N = 29). Yellow circles show localities of either uncertain dating or studies that make no reference to the EOT (N = 24). Palynological sites that only have data for either the Priabonian or the Rupelian are indicated separately (N = 122). Map generated in ArcMap 10.4.1.

**Figure 3:**
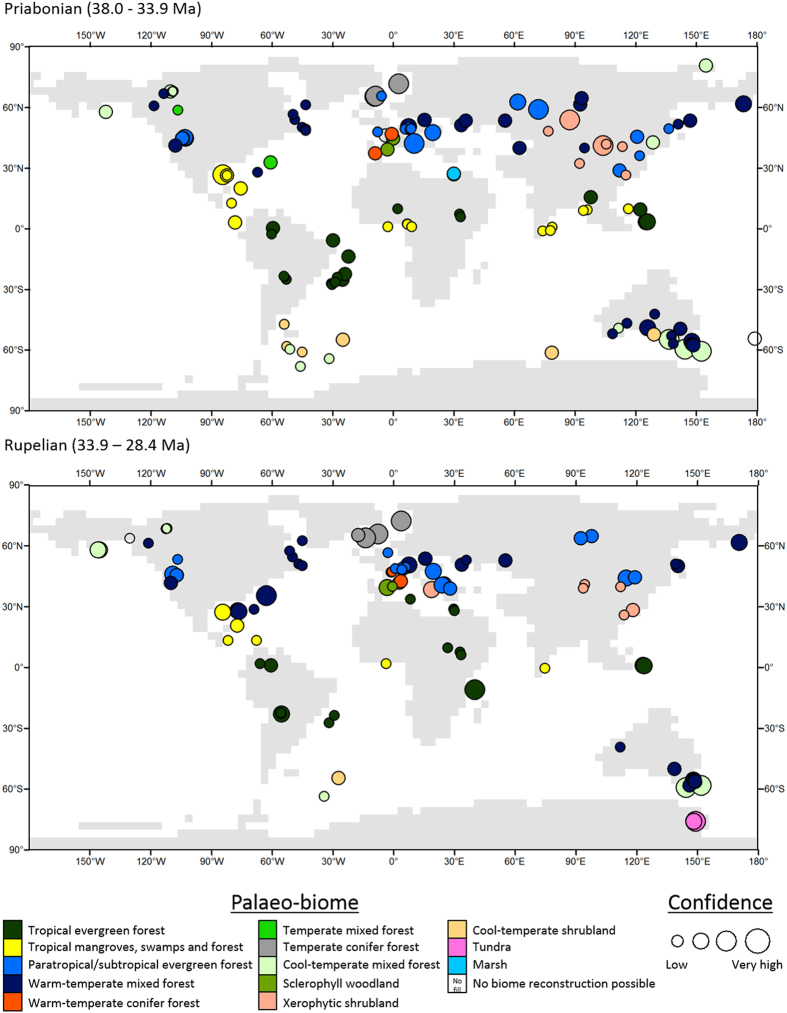
Global palaeo-biome reconstructions for the Priabonian and Rupelian. Palaeo-biomes were reconstructed using the Jaccard Similarity of the presence-absence data of pollen and spore taxa, which were grouped using a cluster analysis and groups were objectively defined using SIMPROF (see methods for full details). Map generated in ArcMap 10.4.1.

**Figure 4:**
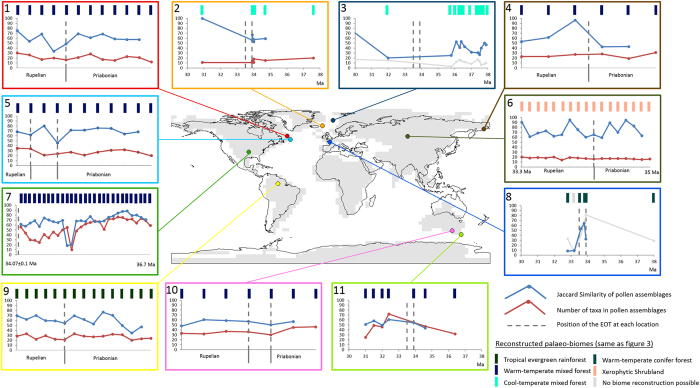
Changes in the Jaccard Similarity of ten pollen records that cross the EOT. The Jaccard Similarity (blue line) is a measure of similarity between a pollen assemblage and the assemblage that is immediately stratigraphically below it. The number of named pollen taxa (red line) provides a simple means to determine if taphonomic processes (preservation/identification of palynomorphs) might have influenced the similarity of pollen assemblages. Some locations have numerical chronologies, whilst others are dated to geological stages (full details of dating techniques applied at each site are presented in Supplementary Table S2). Map generated in ArcMap 10.4.1.

Our data synthesis provides no evidence for a global-scale uniform terrestrial response to a rapid cooling at the EOT^5^. Instead our global palaeo-biome distributions and terrestrial temperature reconstructions point to a more complex pattern of pre-EOT change, isolated changes at the EOT and regions with no change. Our quality assessment indicates that many localities in the high-latitudes of the northern hemisphere showing palaeo-biome changes at the EOT are associated with low-confidence levels (Fig. 3). In the North Atlantic, several very high-confidence sites with temperate conifer forest are present in both the Priabonian and the Rupelian (Fig. 3) indicating no change, whereas only a single low-confidence paratropical/subtropical forest record in the Priabonian indicates a climatic change at the EOT (Fig. 3). This record comes from DSDP 338 and has low-resolution dating based on palynomorph biostratigraphy^38^. Whereas at the very high-confidence site of ODP 913B, the Jaccard similarity shows a change in vegetation before the EOT at 36–35 Ma (Fig. 4) resulting from the loss of pollen from thermophilic plants^29^. This change before the EOT is supported by atmospheric pCO_2_ reconstructions^12^ (Fig. 1) and changes in central European forests^39^,^40^,^41^. At the high-latitudes of North America one low-confidence locality suggests a 5° southward shift of warm-temperate mixed forest by the Rupelian (Fig. 3). This is based upon a single sample and whilst this may indicate an EOT induced shift in high-latitude vegetation the confidence level is very low due to the poor age control^42^. The change from warm-temperate to paratropical/subtropical forest in Siberia is documented by medium-confidence localities (Fig. 3) These have excellent pollen preservation, but the dating resolution only assigns each locality to a geological stage without greater refinement^43^,^44^,^45^. Previous palaeoclimatic work in Siberia reconstructed a Paleogene minimum in MAT at 35 Ma following a long-term cooling from the middle Eocene^46^, which again coincides with recent stomatal based pCO_2_ reconstruction^12^.

In the mid-latitudes of the northern hemisphere our main palaeo-biome changes between the Priabonian and the Rupelian are associated with the distribution arid biomes in southwest Europe and Central to east Asia (Fig. 3). The expansion of arid biomes in these regions is in agreement with previous studies^26^,^47^,^48^. Aridity in central Asia has been present since the early Eocene and is likely the result of tectonic uplift^48^ and subsequent marine regression during the middle to late Eocene^49^, isolating the region from sources of moisture^48^,^49^. Whilst aridity in Europe has been ascribed to alpine uplift and sea-level fall^47^, this has likely been present since the late Eocene as isotopic evidence from Spain shows no change through the late Eocene and early Oligocene^50^, whilst the major floral change starts during the Priabonian^51^. The palaeo-biome distribution and the Jaccard Similarity of the tropics shows no major change between the Priabonian and the Rupelian (Figs 3 and 4). Whilst the reduced number of tropical mangrove, swamp and forest biome localities during the Rupelian, may relate to the estimated 70 m global sea-level fall associated with the growth of the Antarctic ice-sheet^52^.

**Figure 5:**
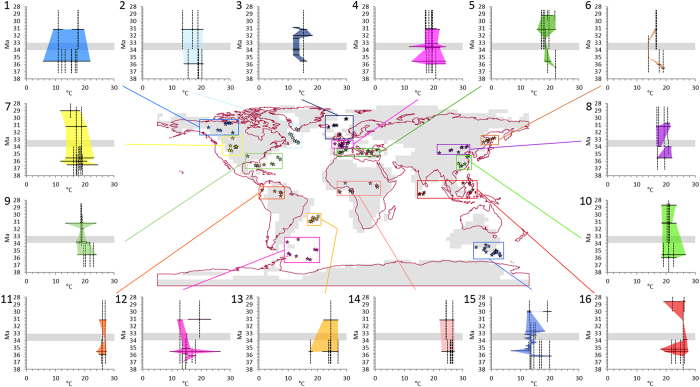
Regional composite (multi-site) Mean Annual Temperature Ranges (MATR) for 16 regions from the beginning of the Priabonian, across the EOT and through the Rupelian. The horizontal bars show the MATR based on the co-existence approach. Vertical bars present the uncertainty ranges for the dating of each pollen assemblage. Colour-shaded areas show the trend in the MATR. Grey bars show the accepted temporal range of the EOT (33.9–33.5 Ma). The MATR has been reconstructed using the co-existence approach, which reconstructs a range of equally possible temperatures within which a fossil floral assemblage could have co-existed^93^ Where a range is narrow there is more confidence in the interpretation of climatic change, whereas a wide range provides lower confidence in our palaeoclimatic interpretations. Map generated in ArcMap 10.4.1.

Palaeo-biome distribution is unchanged between the Priabonian and Rupelian in the southern hemisphere mid-latitudes (Fig. 3). In southern South America terrestrial oxygen isotopes and phytolith analysis have also demonstrated a quasi-static climate and vegetation across EOT^23^,^31^. Conversely, fire dynamics in this region do change before the EOT at ~35 Ma and after the EOT in a short-lived 200 ka humid interval^53^. Coincident with this first change in fire dynamics is the reconstructed cooling of the MATR for the south western Atlantic region by ≤5 °C (Fig. 5). Palaeo-biome distribution remains constant in Australia, whilst the MATR reconstructions shows a cooling event prior to 35 Ma (Figs 3 and 5). The presence of high to very high-confidence tundra palaeo-biome localities on Antarctica demonstrates the continuation of vegetation in the early Oligocene on the continent despite the development of a large Antarctic ice sheet^54^,^55^,^56^.

Much of our prior knowledge of vegetation at the EOT comes from macrofossil syntheses in North America that proposed equatorward shifts in palaeo-biomes^1^,^27^,^57^. Reanalysis of these macrofossil sites has, however, revealed a more gradual cooling through the late Eocene^30^. Other regional reviews of the palaeobotanical evidence have demonstrated that the terrestrial record shows a high degree of heterogeneity in climate and vegetation before, during and after the EOT^1^,^12^,^22^,^27^,^58^. This is in agreement with our global reconstruction based on palynological data and suggests that the terrestrial realm may have undergone a long-term transitional interval that began at the end of the middle Eocene. However, terrestrial oxygen isotopes from North America and northwest Europe have reconstructed MAT decreases of ≤8 °C at the EOT^18^,^19^, which is not in agreement with our palynological results. However, the interpretation of these records is subject to controversy. Further work in North America found no change in MAT but a shift in precipitation^59^, whilst those from northwest Europe may represent a change in the growing season temperature and hydrological source^19^,^60^. The heterogeneity in the timing and scale of change in the vegetation record through the late Eocene–early Oligocene reported here and by others complicates the idea that mammalian turnovers in Europe and Asia were a direct result of vegetation change promoted by EOT cooling^24^,^26^. The Mongolian Re-modelling (MR) in Asia^61^ and the Grande Coupure (GC) in Europe are dated to around the EOT^24^,^51^. The MR is related to increased aridity in central Asia^61^, a result consistent with our palaeo-biome reconstructions showing an increase in xerophytic shrubland in central Asia during the Rupelian (Fig. 3). The GC is associated with a shift to more seasonal vegetation types and the biogeographical spread of Asian mammalian lineages^24^,^51^. Our reconstructions show some spread of more seasonal palaeo-biomes around the Mediterranean, but not in more northern Europe (Fig. 3). Elsewhere in the world, the fossil mammal record shows faunal changes at the end of the middle Eocene and sometimes no response to the EOT^62^,^63^,^64^,^65^. Overall the terrestrial realm shows a pronounced longer-term development from the end of the middle Eocene to the early Oligocene^66^, this is particularly well demonstrated through our synthesis of global palynological records and requires a more complex mechanism than a rapid and large drawdown of CO_2_ at the EOT to explain this global heterogeneity.

Insufficient age control and the lack of modern analogue vegetation are inherent to most deep-time geological studies and also put limitations on our approach that need to be considered before further discussing the wider implications of our findings. The low temporal resolution of our records may prevent the identification of a hypothesised “transitional” EOT with a short-term cooling and a subsequent return to near pre-EOT levels^3^,^16^. However, neither the regional temperature reconstructions (Fig. 5) nor the 11 sediment records of our dataset which have sufficient age control and resolution to identify short term change at the EOT (Fig. 4), support the hypothesis a “transitional” EOT.

We minimized the potential uncertainty of using NLR to reconstruct biomes by applying a statistical approach to produce palaeo-biomes (Fig. 3). This method used presence/absence data rather than abundance of individual pollen taxa. By focussing on presence/absence data we removed any noise in our reconstructions from habitat variation, landscape changes, shifts in depositional environment or the differences between palynologist’s counting techniques^67^. Our approach is specifically designed to identify large changes in biome distribution primarily driven by climate changes and accompanied by distinct changes in taxa composition. However, for this reason our approach cannot detect smaller scale relative changes in abundance, which may explain some differences in our global scale reconstruction when compared to individual studies that found change at the EOT from fluctuations in the proportion of different pollen types^26^.

Whilst our palaeo-biome and MAT reconstructions show no uniform global change across the EOT, there is a wealth of evidence for a marine realm disruption^1^. Some of these could be related to the impacts of a large sea-level fall on shallow marine ecosystems^1^, which is also reflected in the reduction of mangrove palm diversity in Africa during the Rupelian^68^. The Eocene - Oligocene appears to have seen a global scale reorganization of oceanic currents^7^,^69^. Numerous geochemical proxies have recorded a global cooling of surface and deep waters across the EOT which was most pronounced at the high latitudes^3^. Recent evidence from the North Atlantic indicates that bottom water formation could have begun during the middle Eocene^70^ and northern component water entered the South Atlantic before the EOT^71^. Tropical Pacific records show significant inputs of reworked material with erosional hiatuses during the EOT^72^ and deep sea gastropod diversification increases in response to more nutrients entering the deep sea at this time^73^. Diatoms show their first global diversity peak^74^ during the EOT, possibly in response to large surface water productivity changes^75^ and an acceleration of the phosphorous cycle due to alterations in ocean circulation^76^. This interval of changing oceanic currents is supported by model simulations that show an early Oligocene Antarctic ice sheet would have drastically altered Southern Ocean flow^77^ and that deep ocean cooling can be achieved through ocean gateway changes without changing atmospheric pCO_2_^7^. The deep ocean oxygen isotope signal may therefore not necessarily reflect global surface temperature changes at the EOT^7^.

Our study questions previous notions of a uniform, abrupt change of terrestrial vegetation and climate at the EOT and a rapid shift from a greenhouse to an icehouse world. The growth of an Antarctic ice sheet through the late Eocene^78^ that was driven by substantial late Eocene cooling^79^ (Fig. 5) significantly reorganised oceanic circulation^37^. It has been shown that terrestrial sites proximal to the oceans have strong hydrological connections at the EOT^60^, which may mean these were sensitive to changes in ocean currents or sea level fall that were the result of the formation of the Antarctic ice sheet. A strong link between near-coastal and the marine realm explains why one of the regions where we identify an EOT cooling was around the Gulf Coast of North America (Fig. 5). Ongoing orogenic uplift in North America^80^, Europe^47^ and central Asia^81^ would have created rain-shadows and promoted the expansion of seasonal and arid biomes (Fig. 3). Changes in the sources of moisture to the continents^47^,^60^ and the generation of orographic barriers^47^,^80^,^81^ would have had highly regionalised and temporally disparate impacts, which is seen in the global vegetation record. Whilst our results do not show a response to a rapid and large atmospheric pCO_2_ drawdown at the EOT, the North Atlantic, northeast Asia, south western Atlantic and southeast Australia regions all reconstruct a late Eocene high-latitude cooling that coincides with the drop in the stomatal indices based pCO_2_ reconstruction at 36–35 Ma^12^ and the onset of ice-rafted debris in the Weddell Sea^78^. Further work is required to fully understand the late Eocene evolution of the earth system that created the conditions necessary to cause substantial growth of the Antarctic ice sheet at the EOT, without causing significant change in global vegetation in unison with the oxygen isotope excursion.

## Methods

### Palaeo-biome reconstructions

**Figure 6:**
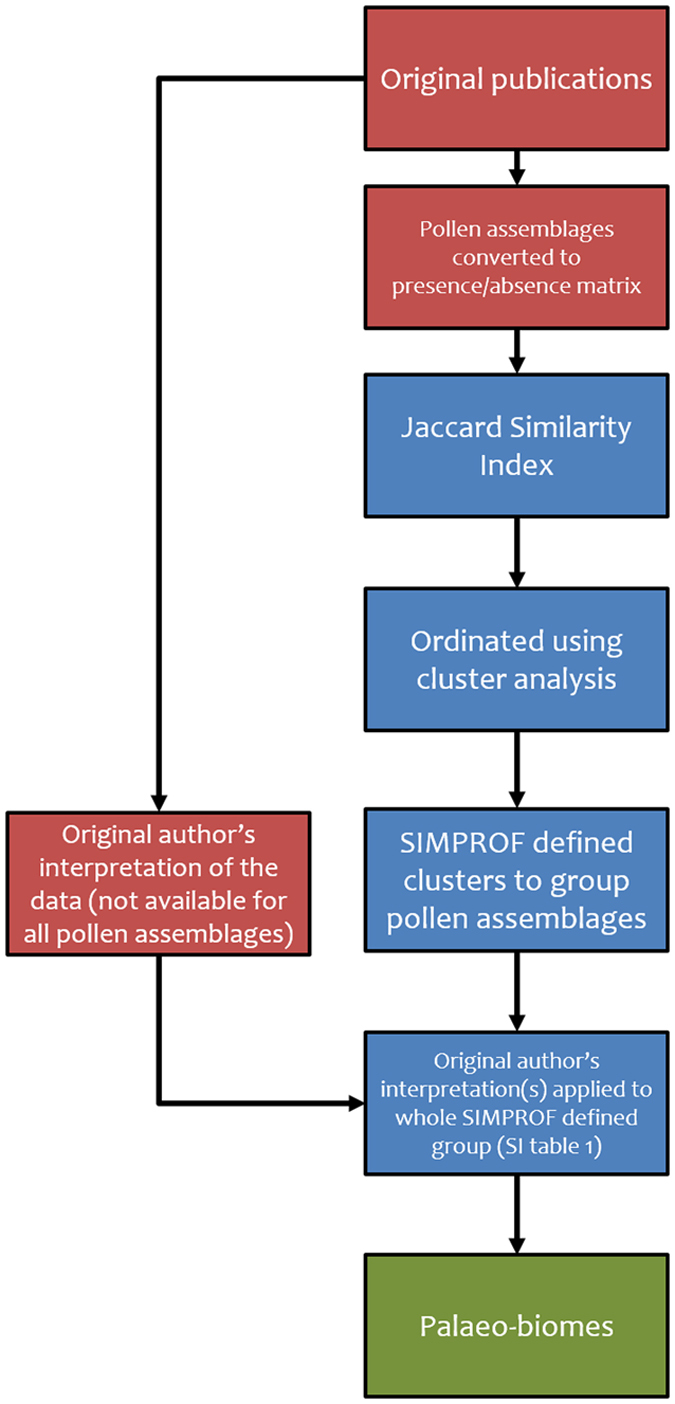
Methodological flow diagram showing the steps taken to produce the palaeo-biomes of the EOT. The flow diagram shows the processes undertaken to develop statistically grouped palaeo-biomes for the EOT. Colours of boxes refer to stages of the process: red = original data; blue = statistical grouping and interpretation; green = end product.

We synthesised 397 pollen assemblages from 216 localities with ages covering the Priabonian to the Rupelian into TEVIS^82^ (Fig. 6). In our analysis we only used plant microfossils (pollen and spores) rather than an approach integrating all plant fossil data (such as has been used in refs 20,21,82), due to the statistical grouping applied to the data. Fossil pollen and spore taxa and macrofossil taxa, do not share a common taxonomy and assigning a fossil pollen grain and a fossil leaf to a parent species is a difficult prospect, except in the rare instances that multiple articulated plant parts are preserved together^83^. Therefore, if plant macrofossils had been included in the statistical analysis there would have been no shared similarity between the two. Whilst a workaround might have been to assign both micro- and macrofossils to genus or family level bins (e.g. all fossils related to *Quercus* assigned to that genus-level bin), this would have artificially increased the similarity and ignored the biogeography of well documented pollen, spore and macrofossil taxa^84^. As research on fossil pollen and spores is far more commonplace (their small size and utilisation for biostratigraphy make them more routinely studied than plant macrofossils), a focus on pollen and spores was determined as the best way of maximising the geographical area covered and producing a large dataset that would enable us address our hypotheses.

The latitude and longitude for each site were converted to palaeo-latitude and palaeo-longitude using the technique presented in Hunter *et al*.^85^. Beside palaeo-coordinates, TEVIS also includes data on the age of the pollen assemblage, sedimentology, method used to date the sample and a quality (Q) indicator to evaluate the dating uncertainties. The Q – indicator applies a qualitative classification to the dating of a pollen assemblage, preservation of the material and original purpose of the publication (palaeoclimate/palaeoenvironmental versus pure biostratigraphical study) where a Q-number of 1 would equal a pollen assemblage with excellent age constraint either through radiometric dating or multiple biostratigraphic frameworks combined with magnetostratigraphy that is well preserved and the original study had a focus on either palaeoenvironments or palaeoclimates; a Q-number of 3 would be a site with one or more biostratigrapical frameworks that are independent of palynology, with moderate to good preservation of the palynomorphs and, whilst the main focus may not have been palaeoenvironmental, some interpretations were made by the original authors and a Q-number of 5 is applied to localities that have been presented with little evidence for how they were dated, or have very poor fossil preservation or contained no information on the palaeoenvironment. 18.6% of the dataset has a quality Q1 or Q2 and only datasets with Q1 to Q4 dating quality were included in this study. Following the methodology of Salzmann *et al*.^21^, we have translated the Q-numbers into a qualitative confidence ranking (low, medium, high or very high). Pollen assemblages had their taxonomic nomenclature standardised using published synonymies^86^,^87^ and the John Williams Index of Palaeopalynology^88^. To avoid artificial similarity, “form genera” without a species diagnosis were excluded from the dataset, as these often have a wide range of botanical affinity (i.e. *Triporopollenites* sp. could originate from Betulaceae, Myricaceae or Proteaceae). Conversely to increase similarity between the pollen assemblages, taxa with different naming traditions, depending on the age of the work and the school of the original author^86^, were combined under a common pollen name (e.g. *Alnus* sp. or *Alnipollenites* sp. as well as named species of these, combined under *Alnus* – type). The pollen assemblages were then filtered to ensure that no pollen assemblage had dating uncertainty that could place it in more than one geological stage (e.g. no individual pollen assemblage was included in the analysis with an age range that covered the Late Eocene and the Early Oligocene). Then the synonymised pollen assemblage data were entered into PRIMER (Plymouth Routines in Multivariate Ecological Research version 6, PRIMER-E Ltd, Plymouth Marine Laboratory, UK^89^,^90^, pre-treated with the presence/absence function and the Jaccard Index Coefficient was applied to generate the resemblance matrix of the pollen assemblages (Fig. 6). The similarity of the pollen assemblages was ordinated as a cluster analysis and similarity profile (SIMPROF) tests were run with 9999 permutations to identify groups of pollen assemblages considered homologous (Fig. 6). SIMPROF seeks to identify clusters within a hierarchical cluster analysis whereby no further structure (branching) can be identified with increasing similarity^91^. These grouped pollen assemblages were then assigned a palaeo-biome based on the interpretations of the original pollen assemblage’s authors (Fig. 6). In all palaeo-biomes with multiple pollen assemblages the different author’s interpretations did not disagree. The palaeo-biomes were then plotted in ArcGIS 10 using a palaeogeographic map from Markwick^92^.

### Pollen assemblages across the EOT

We utilised eleven sites from the TEVIS dataset with a continuous record covering the EOT to analyse pollen assemblage changes through time. These pollen assemblages are confidently tied to a chronostratigraphy that allows the position of the EOT to be known; number 2, 3, 8 & 11 have a numerical chronology; 6 & 7 have dated horizons at the top and bottom of the records; 1, 4, 5, 9 & 10 do not have a numerical chronology, but the position of the EOT is known (Supplementary Table S2). The Jaccard Similarity Index of each pollen assemblage, relative to the pollen assemblage that was immediately stratigraphically below it was then plotted for each site. The number of pollen taxa in the dataset was also plotted, as a significant increase or decrease in the number of pollen taxa would artificially lower the similarity of successive assemblages.

### Terrestrial climatology

We used the Co-existence Approach (CA) to reconstruct the Mean Annual Temperature Range (MATR), Coldest Month Mean Temperature Range (CMMTR) and Warmest Month Mean Temperature Range (WMMTR). The CA reconstructs a range of equally possible values within which a fossil plant community could have co-existed, rather than an absolute value with error margins^93^,^94^,^95^. Whilst some authors have taken the mid-value of the range and plotted these with the highest and lowest values of the range as error bars, this is not recommended as it can lead to spurious uses of reconstructions by non-experts^93^,^94^,^95^. The CA is robust at reconstructing temperature changes of 1 °C^95^, which is sensitive enough to identify the large-scale climate changes proposed by previous estimates of terrestrial temperature changes^18^,^19^. These climatic parameters have been reconstructed for all Eocene – Oligocene pollen assemblages recorded in TEVIS using the Palaeoflora database^93^,^94^,^95^. We have also used the data available from Australia’s Virtual Herbarium^96^ and the Atlas of Living Australia^97^ to define climatic ranges for species identified as nearest living relatives to southern hemisphere Eocene – Oligocene plants. The CA reconstructs possible ranges within which a fossil plant assemblage could have co-existed and has been widely applied to palaeoclimate studies of the EOT and other geological intervals^28^. Locations were grouped into regions based on their palaeo-latitude and palaeo-longitude and then these were plotted chronologically to produce regional MATR, CMMTR and WMMTR reconstructions.

The use of Nearest Living Relatives (NLR) in reconstructing vegetation communities and environmental variables, such as temperature, makes the assumption that modern biomes and species climate tolerances can be extended into the past^95^. This assumption has been justified through multi-proxy studies covering most of the Cenozoic that show comparable results of terrestrial temperature reconstructions using NLR, physiognomic and isotopic methods^95^,^98^. Despite the widespread agreement between different terrestrial temperature reconstruction techniques, we have focussed our results on relative changes in temperature rather than absolute (Fig. 5).

## Additional Information

**How to cite this article**: Pound, M. J. and Salzmann, U. Heterogeneity in global vegetation and terrestrial climate change during the late Eocene to early Oligocene transition. *Sci. Rep.* **7**, 43386; doi: 10.1038/srep43386 (2017).

**Publisher's note:** Springer Nature remains neutral with regard to jurisdictional claims in published maps and institutional affiliations.

## Supplementary Material

Supplementary Information

Supplementary Dataset 2–4
