# Supplementary material for: Heterogeneity in global vegetation and terrestrial climate change during the late Eocene to early Oligocene transition
Source: Sci Rep. 2017 Feb 24;7:43386. doi: 10.1038/srep43386 (PMC5324063; doi:10.1038/srep43386)
Supplement: Supplementary Information [file srep43386-s1.doc]

**Heterogeneity in global vegetation and terrestrial climate change during the late Eocene to early Oligocene transition**

Matthew J. Pound and Ulrich Salzmann

**Supplementary information**

**Supplementary Figure S1.** Cluster analysis of the Jaccard similarity of the EOT pollen assemblages. The green dashed lines are clusters that are defined as homogeneous by SIMPROF and have been closed to fit the graph onto a page. Each closed cluster has been assigned a letter(s) in parentheses and these correspond to supplementary information table 1, where details of the closed clusters are provided. The red dashed lines are SIMPROF defined clusters that have not been closed. Coloured dots show the palaeo-biome assigned to each sample; palaeo-biome assignment to the closed clusters is available in supplementary information table 1.

**Supplementary Table S1**. Details of the SIMPROF defined clusters. Including which pollen assemblages were included in that cluster, the similarity level of the whole cluster, the palaeo-biome of that cluster and the references for the palaeo-biome determination. Pollen assemblage numbers refer to supplementary Table S2 and those with an underscore were used for the higher resolution Jaccard similarity plots (Fig. 4).

| Cluster | Layers in cluster | Cluster Similarity level (%) | Palaeo-biome | References for palaeobiome assignment |
| --- | --- | --- | --- | --- |
| A | 210, 169, 170, 8, 282, 283 | 18.5 | Cool-temperate shrubland | Macphail and Truswell, 2004 |
| B | 256, 59, 145, 149, 244, 245, 11, 336, 249, 249_36.4, 249_31, 254, 13, 14, 255, 253, 249_34.6, 251, 249_33.9, 249_32.4, 252, 249_32, 249_31.5, 250, 35, 33_1, 33_4, 33, 33_2, 33_3, 34, 33_5, 33_6 | 19 | Warm-temperate mixed forest | Truswell and Marchant, 1986; Martin, 1997; Macphail and Stone, 2004; Macphail, 2007 |
| C | 27, 324 | 50 | Tundra | Francis et al., 2008 |
| D | 325, 326, 327, 12, 271, 184, 339, 321, 322, 323 | 25 | Cool-temperate mixed forest | Fasola, 1969; Evitt and Pierce, 1975; Romero and Zamaloa, 1997; Grube and Mohr, 2007 |
| E | 277, 318, 316, 317, 319 | 11 | Tropical evergreen forest | Graham, 2010; Lelono and Morley, 2011 |
| F | 246, 247 | 22 | Tropical mangroves, swamps and forests | Graham, 1985 |
| G | 164, 162, 160, 161, 163 | 17 | Tropical evergreen forest | Eisawi and Schrank, 2008 |
| H | 284, 276, 38, 39, 40, 41, 278, 279, 72, 190, 66_3, 66_7, 66_10, 67, 66_1, 66_4, 66_5, 66_6, 66_9, 66, 68, 66_13, 66_12, 66_14, 66_8, 66_15, 66_2, 66_11 | 15 | Tropical evergreen forest | Dueñas-Jimenez, 1986; Maizatto, 2001; Rull, 2003; Graham, 2010 |
| I | 262, 167, 165, 166, 155, 153, 154, 105, 168 | 15 | Tropical mangroves, swamps and forests | Fong and Said, 2002; Atta-Peters and Salami, 2004; Digbehi et al., 2012 |
| J | 151, 158, 159 | 11 | Tropical evergreen forest | El-Sabrouty, 1998 |
| K | 22, 23, 17, 15, 16, 50, 280 | 9 | Tropical mangroves, swamps and forests | Venkatachala and Rawat, 1973; Mandal, 1997; Saxena and Trivedi, 2009 |
| L | 156, 157 | 5 | Marsh | Kedves, 1986 |
| M | 117, 118 | 16 | Paratropical/subtropical mixed forest | Rusu et al., 1993 |
| N | 204, 206, 205, 207 | 84 | Tropical mangroves, swamps and forests | Tomasini-Ortiz and Martinez-Hernandez, 1984 |
| O | 174, 177, 175, 179, 176, 178 | 10 | Tropical evergreen forest | Graham, 2010 |
| P | 77, 78, 79, 80 | 27 | Warm-temperate mixed forest | Arkhipov et al., 2005; Volkova and Kuz'mina, 2005 |
| Q | 229, 230 | 10 | Tropical mangroves, swamps and forests | Yancey et al., 2003 |
| R | 287, 340, 103, 132 | 26 | Warm-temperate conifer forest | Châteauneuf, 1980; Pais, 1992; Collinson and Hooker, 2003 |
| S | 19, 44, 259, 260 | 6 | Xerophytic shrubland | Lei, 1985 |
| T | 58, 228, 226, 227 | 13 | Paratropical/subtropical mixed forest | Long and Sweet, 1994 |
| U | 133, 266 | 13 | Warm-temperate conifer forest | Châteauneuf, 1980; Châteauneuf and Nury, 1995; Collinson and Hooker, 2003 |
| V | 102, 106, 335, 109, 110, 116, 10, 115, 331, 332, 334_27, 333_31, 334_26, 334_25, 333_35, 333_30, 333_28, 333_34, 333_29, 333_33, 333_32 | 12 | Xerophytic shrubland | Yu et al., 2003; Yang et al., 2005; Fortuna and Erfurt, 2006; Guo et al., 2006; Lu et al., 2010; Hoorn et al., 2012 |
| W | 31, 32, 83, 107, 108, 84, 29, 30 | 17 | Warm-temperate mixed forest | Mikhelis and Uziyuk, 1973; Lopatina, 2001 |
| X | 212, 216, 217, 224, 222, 215, 223, 214, 225, 218, 213, 211, 219, 220, 221 | 17 | Paratropical/subtropical mixed forest | Newman, 1981 |
| Y | 306, 311, 294_9, 294_10, 294_2, 294_1, 294_8, 294_4, 294_7, 294_5, 299_1, 294_3, 299_2, 294_6, 299_4, 299_3, 299_5, 294_11, 294_12, 299_8, 299_9, 299_10, 303_11, 304, 315, 299_6, 299_7, 314, 305, 310, 88_2, 88, 88_1, 88_6, 89, 88_5, 88_3, 88_4, 131, 264, 265, 286, 233, 234 | 20 | Warm-temperate mixed forest | Hopkins Jr. and Norris, 1974; Williams, 1986 |
| Z | 199, 289, 330, 26, 76, 75, 267, 268 | 19 | Paratropical/subtropical mixed forest | Arkhipov et al., 2005; Akgün et al., 2012 |
| AA | 112, 193, 194 | 17 | Paratropical/subtropical mixed forest | Freshney et al., 1982; Collinson and Hooker, 2003 |
| AB | 114, 275, 274, 272, 273 | 23 | Warm-temperate mixed forest | Guo et al., 2006; Slodkowska, 2009 |
| AC | 329, 241, 242, 243, 238, 237, 120, 127, 130, 121, 124, 240, 119, 125, 122, 123, 128, 126, 129, 9, 28 | 16 | Cool-temperate mixed forest | Norris, 1997; Yi et al., 2003 |
| AD | 263, 5_32, 5_31.8, 6_37.6, 6, 6_35.8, 5, 5_32.4, 6_37.4, 6_36.6, 6_36.3, 6_38, 6_36.1, 6_29.7, 6_32, 7, 5_31.3, 6_37.8, 6_37, 6_37.9, 1_34a, 4, 1_33.9a, 1_30.9, 1_34, 5_31.6, 6_36.4, 5_31.2, 5_31, 1_33.9, 3, 5_31.5, 1_37.6, 5_30.8, 6_37.5, 2, 1, 1_34.7 | 11 | Temperate conifer forest | Eldrett et al., 2009 |
| AE | 86, 87 | 18 | Warm-temperate mixed forest | Blumenstengel et al., 1996 |
| AF | 96, 97, 46, 47, 198, 111, 197, 49, 48, 92, 200, 93, 98, 99 | 41 | Paratropical/subtropical mixed forest | Gruas-Cavagnetto and Barbin, 1989; Konzalová, 1990; Ermolli, 1991; Knobloch and Konzalová, 1998 |
| AG | 136, 140_38, 137, 328, 138, 135, 134, 139, 140_32.8, 140, 140_33.6, 140_33.9, 140_33.8 | 15 | Warm-temperate conifer forest | Châteauneuf, 1980 |
| AH | 113, 285 | 24 | Sclerohphyll woodland | Ruiz, 1987 |
| AI | 269, 270 | 12 | Sclerohphyll woodland | Cavagnetto and Anadón, 1996 |
| AJ | 73, 74, 257, 90, 91, 64, 94 | 31 | Paratropical/subtropical mixed forest | He and Sun, 1977; Liu, 1987; Zhang, 1988; Gengwu and Rongyu, 1999 |
| AK | 337, 338 | 7 | Tropical mangroves, swamps and forests | Gennet, 1996 |
| AL | 61, 62, 63, 236, 235_1, 235_2 | 29 | Warm-temperate mixed forest | Frederiksen, 1980; Oboh and Morris, 1994; O' Keefe et al., 2005 |
| AM | 208, 209, 202, 203 | 24 | Tropical mangroves, swamps and forests | Frederiksen, 1988; Gennet, 1996 |
| AN | 231_18, 231_17, 231_11, 231_20, 231_16, 231_19, 231_10, 231_14, 231_15, 231_9, 231_13, 231_3, 231_4, 231_8, 231_7, 231_5, 231_6, 231_1, 231_2, 231_12, 232_8, 232_6, 232_4, 232_2, 232_5, 231_21, 232_7, 232_1, 232_3 | 18 | Warm-temperate mixed forest | Wingate and Nichols, 2001 |

**Coldest month and warmest month mean temperatures**


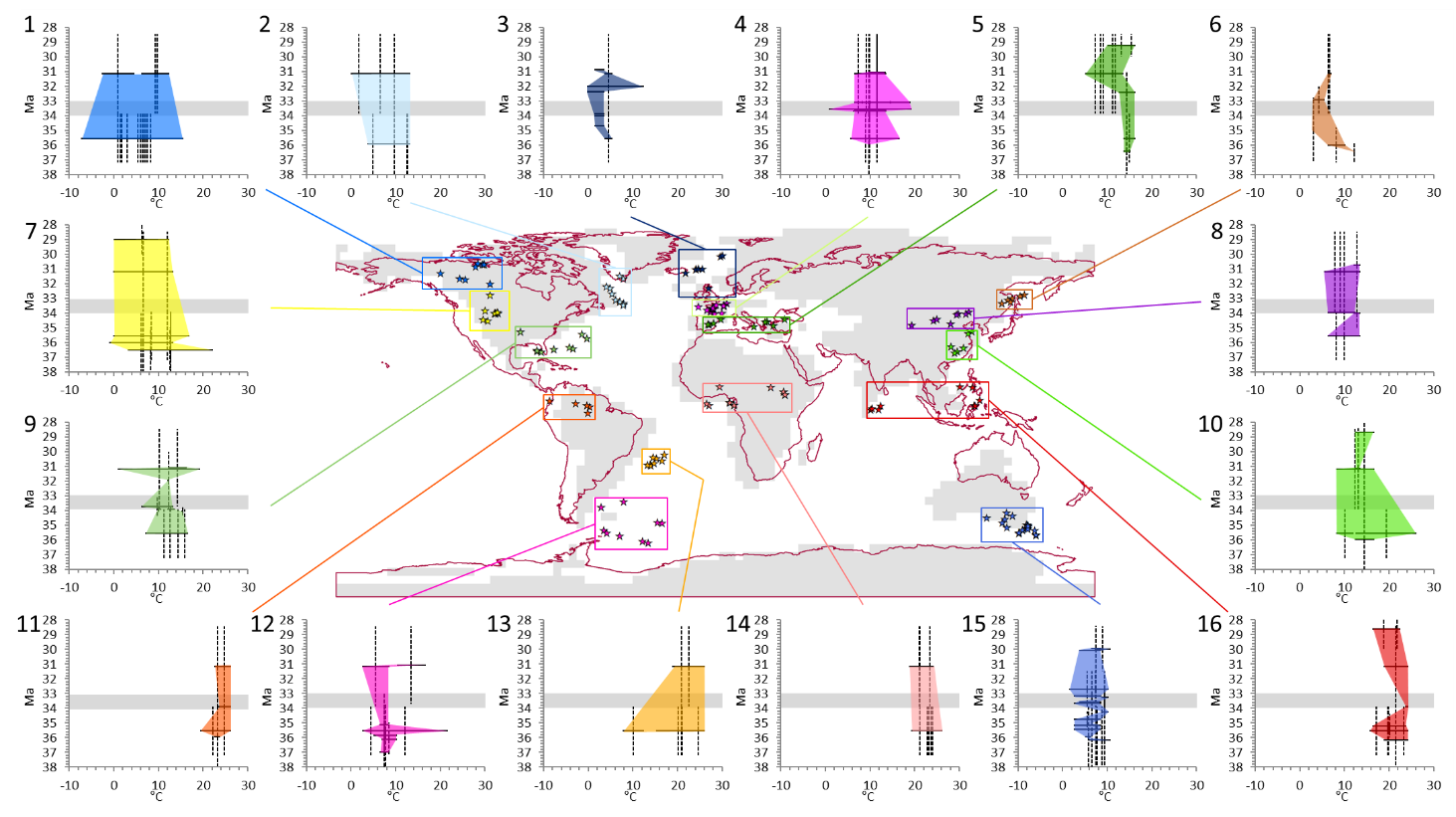


**Supplementary Figure S2:** Regional composite (multi-site) Cold Month Mean Temperature Ranges (CMMTR) for 16 regions from the beginning of the Priabonian, across the EOT and through the Rupelian. *The horizontal bars show the CMMTR based on the co-existence approach. Vertical bars present the uncertainty ranges for the dating of each pollen assemblage. Colour-shaded areas show the trend in the CMMTR. Grey bars show the accepted temporal range of the EOT (33.9 – 33.5 Ma). The MATR has been reconstructed using the co-existence approach, which reconstructs a range of equally possible temperatures within which a fossil floral assemblage could have co-existed^98^ Where a range is narrow there is more confidence in the interpretation of climatic change, whereas a wide range provides lower confidence in our palaeoclimatic interpretations. Map generated in ArcMap 10.4.1.*


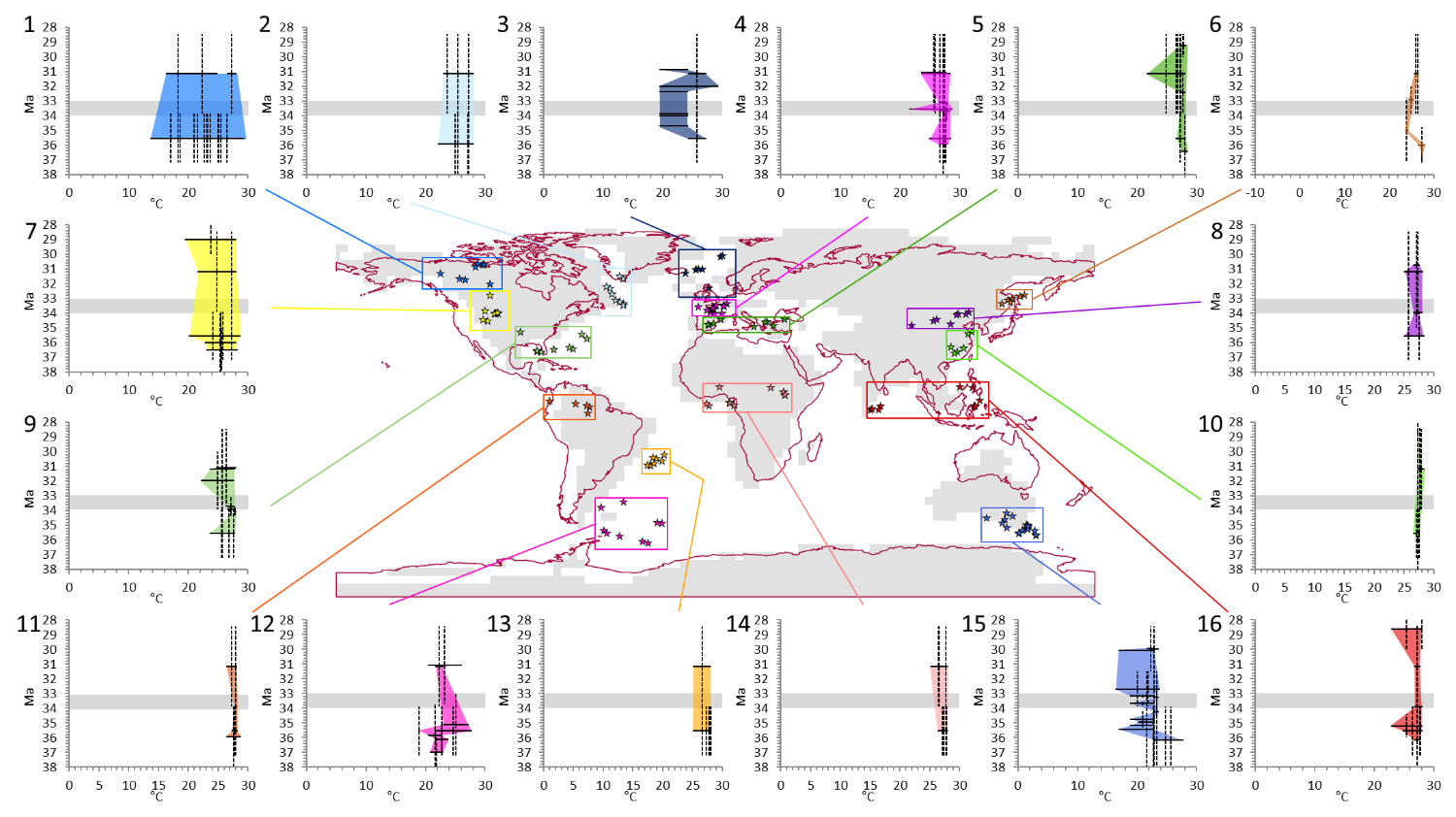


**Supplementary Information Figure 3:** Regional composite (multi-site) Warm Month Mean Temperature Ranges (WMMTR) for 16 regions from the beginning of the Priabonian, across the EOT and through the Rupelian. *The horizontal bars show the WMMTR based on the co-existence approach. Vertical bars present the uncertainty ranges for the dating of each pollen assemblage. Colour-shaded areas show the trend in the WMMTR. Grey bars show the accepted temporal range of the EOT (33.9 – 33.5 Ma). The MATR has been reconstructed using the co-existence approach, which reconstructs a range of equally possible temperatures within which a fossil floral assemblage could have co-existed^98^ Where a range is narrow there is more confidence in the interpretation of climatic change, whereas a wide range provides lower confidence in our palaeoclimatic interpretations. Map generated in ArcMap 10.4.1.*
